# Hepatic Artery Pseudoaneurysm in the Liver Transplant Recipient: A Case Series

**DOI:** 10.1155/2019/9108903

**Published:** 2019-12-27

**Authors:** David P. St. Michel, Naeem Goussous, Nathalie L. Orr, Rolf N. Barth, Stephen H. Gray, John C. LaMattina, David A. Bruno

**Affiliations:** Department of Surgery, University of Maryland School of Medicine, Baltimore, MD, USA

## Abstract

**Introduction:**

Hepatic artery pseudoaneurysm is a rare and potentially fatal complication of liver transplantation with a reported incidence of 0.3–2.6% and associated mortality approaching 75%. Clinical presentation typically includes sudden hypotension, gastrointestinal bleed or abnormal liver function tests within two months of transplantation. We report a series of four cases of hepatic artery pseudoaneurysm in adult liver transplant recipients with the goal of identifying factors that may aid in early diagnosis, prior to the development of life threatening complications.

**Methods:**

A retrospective chart review at a high volume transplant center revealed 4 cases of hepatic artery pseudoaneurysm among 553 liver transplants (Incidence 0.72%) between March 2013 and March 2017.

**Results:**

Two of the four patients died immediately after intervention, one patient survived an additional 151 days prior to death from an unrelated condition and one patient survived at two years follow up. All cases utilized multiple imaging modalities that failed to identify the pseudoaneurysm prior to diagnosis with computed tomography angiography (CTA). Two cases had culture proven preoperative intrabdominal infections, while the remaining two cases manifested a perioperative course highly suspicious for infection (retransplant for hepatic necrosis after hepatic artery thrombosis and infected appearing vessel at reoperation, respectively). Three of the four cases either had a delayed biliary anastomosis or development of a bile leak, leading to contamination of the abdomen with bile. Additionally, three of the four cases demonstrated at least one episode of hypotension with acute anemia at least 5 days prior to diagnosis of the hepatic artery pseudoaneurysm.

**Conclusions:**

Recognition of several clinical features may increase the early identification of hepatic artery pseudoaneurysm in liver transplant recipients. These include culture proven intrabdominal infection or high clinical suspicion for infection, complicated surgical course resulting either in delayed performance of biliary anastomosis or a biliary leak, and an episode of hypotension with acute anemia. In combination, the presence of these characteristics can lead the clinician to investigate with appropriate imaging prior to the onset of life threatening complications requiring emergent intervention. This may lead to increased survival in patients with this life threatening complication.

## 1. Introduction

Hepatic artery pseudoaneurysm (HAPA) is a rare and potentially fatal complication of liver transplantation [1–12]. Reported incidence ranges between 0.3% and 2.6%, with associated mortality reported as high as 75% [1–12]. While the precise pathogenesis is not fully understood, it is likely a combination of surgical vascular manipulation at the arterial anastomotic site and localized infection in the setting of a compromised immune system [1–12]. Other factors, such as surgical complications, reoperations, bile leak and interventional procedures [1–3, 6] may also play a role in the development of HAPA.

The timing of HAPA presentation is variable. It is most frequently diagnosed within 60 days of transplant [1–12] but may not be recognized until years later [3, 6, 10]. Clinical presentation typically includes a combination of severe abdominal pain, gastrointestinal bleeding, or hemodynamic instability as the HAPA is unrecognized until rupture [1–12]. Elevated liver function tests secondary to obstructive graft dysfunction caused by an unruptured HAPA may also be present [9, 11].

A high clinical index of suspicion, rapid recognition [1–12] and radiographic confirmation of HAPA is necessary for successful treatment. While computed tomography (CT), magnetic resonance imaging (MRI) and doppler ultrasonography can be useful for detection of suspected HAPA, angiography is required for definitive diagnosis apart from diagnosis at the time of laparotomy [1–3, 6, 9–11]. Treatment options include embolization, stenting, thrombin injection, surgical ligation or surgical excision [1–5, 11]. We report a series of four cases of hepatic artery pseudoaneurysm in adult liver transplant recipients with the goal of identifying factors that may aid in early diagnosis of HAPA prior to the development of life threatening complications.

## 2. Methods

A retrospective chart review at a single high volume transplant center was undertaken. All liver transplants between March 2013 and March 2017 were identified. During this period of time our center performed only adult liver transplantation. A total of 553 cases of liver transplantation (including multiorgan transplant) were identified. This revealed four cases of HAPA, an incidence of 0.72%. These charts were further reviewed to determine the clinical course of the patients and to identify potential common characteristics between the cases. Data on the etiology of hepatic failure, preoperative conditions, surgical course, donor characteristics, presentation of HAPA, time to diagnosis of HAPA, methodology of diagnosis and treatment were collected. This study was reviewed and approved by our institutional review board.

## 3. Results

### 3.1. Donor and Recipient Demographics

Among 533 liver transplant recipients we identified four cases of HAPA. Three of the four patients were male; ages at time of transplant were 37, 55, 57, and 62. Model for End-Stage Liver Disease (MELD) scores were 28, 37, 38, and 40. The etiology of liver disease was autoimmune hepatitis, alcohol cirrhosis, hepatitis C cirrhosis and one patient with both hepatitis C and alcohol cirrhosis. All four patients were hospitalized prior to transplantation, with decompensating events including gastrointestinal bleed, spontaneous bacterial peritonitis (SBP), altered mental status, acute renal insufficiency, and recurrent clostridium difficle infection. One patient underwent a simultaneous liver kidney (SLK) transplant, while three had an orthotopic liver transplant (OLT). One of the three patients was a retransplant after necrosis of a previous graft.

All patients received grafts from deceased donors, with two donation after cardiac death (DCD) livers and two donation after brain death (DBD) livers. One patient who initially received a DCD graft was retransplanted with a DBD liver secondary to liver necrosis. One graft possessed a replaced right hepatic artery that was reconstructed ex vivo by anastomosis of the superior mesenteric artery to the celiac artery, while the other grafts had standard anatomy. The respective cold ischemic times for the liver transplants were two hours 34 minutes, three hours five minutes, four hours two minutes and four hours seven minutes. Ages and cause of death of donors were as follows: 22 year old with head trauma; 45 year old with a central nervous system tumor; 32 year old with head trauma and 60 year old with anoxia after a cardiac event.

### 3.2. Diagnosis

All four patients had evidence of infection postoperatively. Two patients had positive intraabdominal fluid cultures (vancomycin resistant *Enterococcus* and *Stenotrophamonas maltophilia*) and two had evidence of infected operative fields at the time of reoperation. None had proven active infections prior to transplant.

Two patients had complicated operative courses during transplantation, both received >30 units of packed red blood cells perioperatively. These patients were left with an open abdomen and biliary anastomosis deferred to a later operation after resuscitation in the intensive care unit. One of these patients was found to have a perforated gallbladder, dense adhesions and inflamed tissues, leading to diffused bleeding and intraoperative cardiac-shock requiring resuscitation. The other patient was a previous transplant recipient who required explantation for necrosis and was anhepatic at time of retransplantation. Our practice is to perform a delayed biliary anastomosis in patients who are unstable intraoperatively. We believe that forgoing the biliary anastomosis in these patients allows for greater mobility of the liver to examine the retroperitonium for bleeding, and that biliary anastomoses completed in these situations are more likely to have complications such as stricture. These patients have a drain tied into the bile duct and are resuscitated in the intensive care unit (ICU) for 24–48 hours before return to the operating room for inspection, biliary anastomosis completion and abdominal closure. One additional patient suffered a bile leak postoperatively.

Our center does not have a protocol for postoperative surveillance ultrasound (US), however the vast majority of recipients, and all the patients in this study, had a postoperative day (POD) one US. This decision is made by the operative team after considering several factors including the intraoperative condition of the donor and recipient artery, presence nonstandard vascular anatomy and measured hepatic artery flow after completion of anastomosis. Follow up US or computed tomography angiogram (CTA) plans for continued monitoring is determined by an interdisciplinary rounding team based on intraoperative findings, initial imaging results and changes in laboratory results or clinical condition. In this case series our patients underwent two, three, five, and five US prior to diagnosis of HAPA. One of these patients had blunted hepatic artery waveforms on repeated US, two had small stable fluid collections in the gallbladder fossa or near the hepatic artery anastomosis and one had no abnormalities noted on imaging.

**Figure 1:**
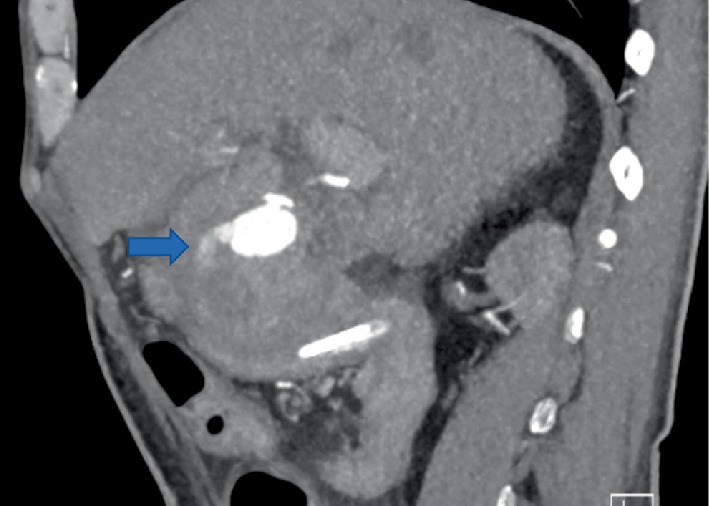
CTA case 1, performed POD #85 showing hepatic artery pseudoaneurysm with active extravasation (arrow).

**Figure 2:**
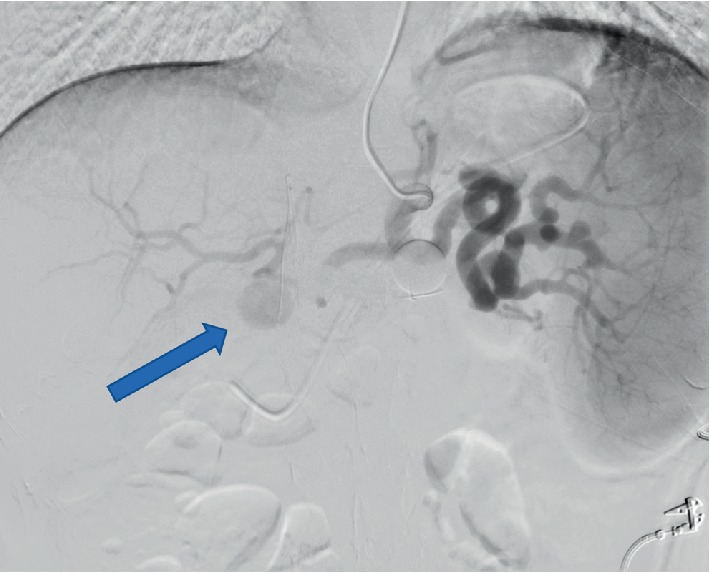
Angiography case 1, performed POD #85 showing hepatic artery pseudoaneurysm (arrow).

**Figure 3:**
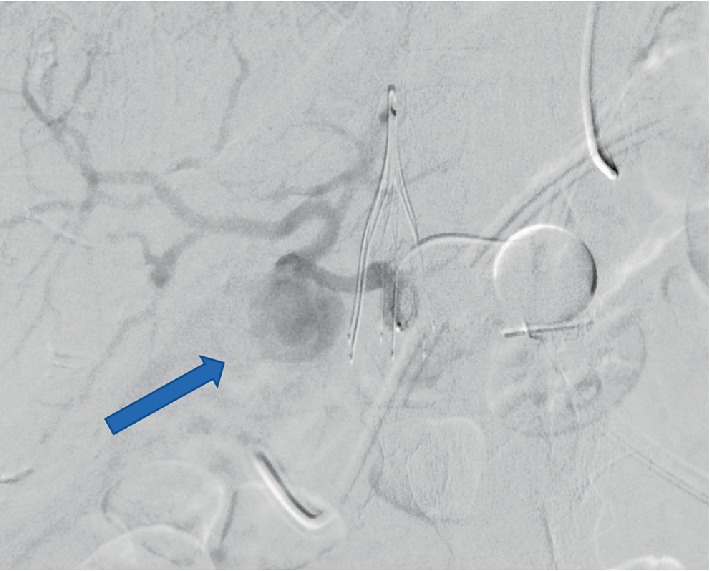
Angiography case 2, performed POD #54 showing hepatic artery pseudoaneurysm with active extravasation (arrow).

All four patients experienced at least one episode of hypotension and acute anemia, with the first episode occurring at a median of 37 (range 5–49) POD. Various imaging modalities were utilized to investigate the cause of these episodes (Figures 1–3). All four were definitively diagnosed with CTA, with an interval from initial hypotensive/acute anemic episode to diagnosis of HAPA of 0, 5, 10, and 54 days. Of the three patients with diagnosis at days 5, 10, and 54, an additional acute hypotensive/anemic episode prompted further investigation and diagnosis.

### 3.3. Management and Outcomes

At the time of diagnosis three patients exhibited rupture of the HAPA. One patient (survival at 24 months follow up with graft function) did not demonstrate rupture and was taken for operative intervention three days after diagnosis, following further characterization of the HAPA in interventional radiology. Of the three patients with ruptured HAPA, two were initially treated with Interventional Radiology (IR) embolization of the HAPA. One of these patients expired from hemorrhage 4 days after the embolization, the other underwent operative intervention the day after IR and expired from massive liver necrosis and hemorrhage two days later. The remaining patient initially underwent operative intervention followed by IR embolization the following day. This patient survived until post-operative day 195, with the cause of death unrelated to the OLT.  Table 1 contains a summary of the clinical course.

In summary, one of the four patients with HAPA has survived to 24 months with a functioning graft. One patient expired secondary to an unrelated procedure (complications of a central line placement) with a functioning graft at 195 days post-transplant. The cause of death of the remaining two patients were hemorrhage and liver necrosis, at 89 and 32 days post-transplant, respectively.

## 4. Discussion

Hepatic artery pseudoaneurysm post liver transplant is a rare but potentially devastating complication. Our retrospective chart review revealed 4 cases of pseudoaneurysm of the hepatic artery among 553 liver transplants (incidence 0.72%), consistent with reported incidence of 0.3–2.6% [1–12]. Two of the four patients died immediately after intervention, one patient survived an additional 151 days (POD #195 from transplantation) prior to death from an unrelated procedure, and one patient survived at two years follow up.

Two cases had culture proven perioperative intrabdominal infections, while the remaining two cases manifested a perioperative course highly suspicious for infection (retransplant for hepatic necrosis after hepatic artery thrombosis and clinically evident infection at reoperation, respectively). Three of the four cases either had a delayed biliary anastomosis or development of a bile leak, leading to contamination of the abdomen with bile. For patients who are unstable intraoperatively or demonstrate continued hemorrhage after repeated attempts at hemostasis, our practice is to leave the abdomen open with a fenestrated plastic barrier and negative pressure dressing. A pediatric feeding tube is placed into the donor bile duct to control biliary output. The patient is then taken to the surgical intensive care unit for resuscitation and correction of coagulopathy, before return to the operating room for abdominal inspection, potential closure and completion of biliary anastomosis. This usually occurs 24–48 hours after initial surgery. In this series, these cases also involved massive transfusion of blood products perioperatively and multiple returns to the operating theater. This complicated operative course should increase the suspicion of HAPA development.

Identification of HAPA prior to rupture may lead to early treatment in a nonemergent setting and therefore a mortality rate lower than the reported 75% [2, 4–6]. Indeed, in our study the patient with survival at 24 months was the only case not found to have active extravasation on CTA. The presentation of HAPA post liver transplant typically occurs within 60 days; the reported mean days to presentation post-transplant ranges between 27 and 35 days, with outlying cases presenting as long as several years post-transplant [1–12]. This is consistent with our mean POD for diagnosis of 49.25 days, with a range of 15–84 days.

Our case series suggests several factors that may aid in early diagnosis of HAPA. First is culture proven or clinically evident infection, which was present in all four of our cases. In six retrospective case reports, which collectively span 1982–2011 and describe 40 experiences of HAPA after liver transplantation, 33 had positive culture data from either blood, surrounding abdominal fluid collection, or aneurysmal wall tissue [2, 4–6, 11, 12]. The presence of preexisting spontaneous bacterial peritonitis (SBP), necrotic tissue or gross contamination at the time of transplant should increase surgical team's concern about the possible development of HAPA [2, 4–6, 11, 12]. Additionally, our series suggests that bile duct complications, either delayed completion of the biliary anastomosis (also indicative of a complicated case) or a bile leak should heighten suspicion of HAPA development [3, 6, 10].

Our case series also revealed that all four patients experienced acute anemia and hypotension—three cases exhibited this clinical scenario at least 5 days prior to definitive diagnosis of HAPA. These instances were treated with blood products and imaging to evaluate for a source of potential hemorrhage, and may represent an initial rupture of the HAPA. All four cases had multiple imaging modalities utilized prior to obtaining a CTA which diagnosed the HAPA. Of the three cases that experienced multiple acute anemic and hypotensive episodes, no CTA was obtained at the index episode. Instead the patients were treated with blood products, US along with CT without contrast were obtained, and the patients stabilized. None of these ultrasounds revealed a HAPA. This suggests a window period between initial symptomatic presentation of HAPA and CTA diagnosis may exist. Recognition of the above clinical scenario should prompt the clinician to obtain a CTA to identify HAPA prior to emergent decompensation of the patient.

**Figure 4:**
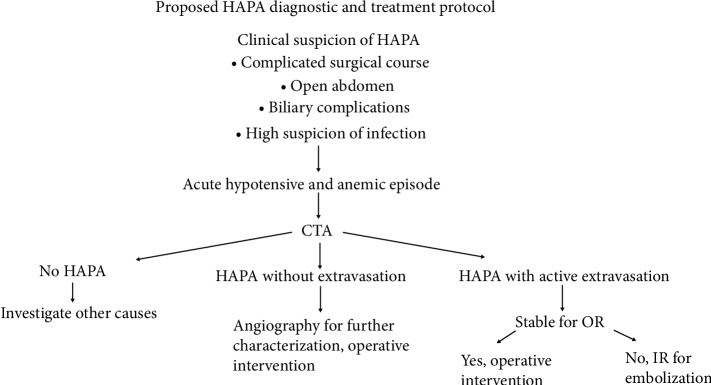
Proposed diagnostic and treatment protocol for HAPA after liver transplant. This emphasizes early CTA to identify HAPA if high clinical suspicion. This potentially would identify HAPA in stable patients, allowing for planned surgical intervention and improved survival.

Notably, three of our cases required emergent intervention in the setting of active hemorrhage. All three of these cases resulted in mortality (one secondary to a separate procedure on POD #195 after a complicated course). This emphasizes the importance of identification of HAPA prior to decompensation, allowing for early surgical intervention. A proposed diagnosis and treatment pathway is presented in Figure 4. Both of the patients whose cause of death was directly related to the liver transplant, first underwent emergent embolization with interventional radiology. The two patients without death related to the transplantation were both initially treated with operative intervention. These patients were considered stable enough to undergo this operative intervention. The lone surviving patient underwent surgical intervention without active hemorrhage, and has survived with normal liver function for 24 months.

Our case series of four patients is too small to support any definitive conclusions regarding the diagnosis and treatment of HAPA. It does, however, suggest several strategies that may prove useful for improving the survival of these patients. A high index of suspicion based on the likelihood of perioperative infection, complicated surgical course, high transfusion requirement, delayed closure of abdomen and biliary complications may aid the clinician in identifying patients at risk of HAPA development. Each of our patients were definitively diagnosed with HAPA through imaging with CTA. In the correct clinical setting, such as acute hypotension and anemia, this test should be obtained initially instead of other imaging modalities. Finally, the two patients who did not die secondary to the liver transplantation both underwent operative intervention initially after HAPA diagnosis. This may have been related to their stability and ability to tolerate a return to the operating room, further suggesting that early diagnosis prior to decompensation is critical for improving patient survival. As this is a rare complication, large multicenter studies will likely be required to confirm these observations.

## 5. Conclusion

Recognition of several clinical features may assist in the early identification of hepatic artery pseudoaneurysm in liver transplant recipients. These include indications of likely perioperative infection, complicated surgical course, high transfusion requirement, delayed abdominal closure and delayed completion of biliary anastomosis or a biliary leak. An episode of hypotension with acute anemia may be a clinical manifestation of the presence of a HAPA. This episode may precede acute decompensation of the patient by days, allowing the clinician the opportunity to investigate with appropriate imaging prior to the onset of life threatening complications requiring emergent intervention. In the correct clinical setting the initial imaging modality should be CTA. Early operative intervention under controlled conditions will likely lead to decreased mortality in these patients.

## Figures and Tables

**Table 1:** Summary of characteristics of hospital course for each case. Infection defined as clinically evident during operation or culture proven.

Characteristic	Case 1	Case 2	Case 3	Case 4
Infection present	Yes, culture proven	Yes, noted intraoperatively	Yes, culture proven	Yes, necrotic tissue in operative field
Patient with open abdomen post transplant	No	No	Yes	Yes
Biliary complication	Yes, bile leak	No	Yes, biliary anastomosis deferred	Yes, biliary anastomosis deferred
Acute anemia (POD)	Yes (30,84)	Yes (13,49,54)	Yes (44)	Yes (5, 10, 29)
Hypotension (POD)	Yes (30,84)	Yes (49,54)	Yes (44)	Yes (5, 10, 29)
Days to diagnosis (POD)	84	54	44	15
Days from hypotensive/acute anemia episode to diagnosis	54	5	0	10
Diagnostic modality	CTA	CTA	CTA	CTA
Mortality (POD)	Yes (89)	No (24 months)	Yes (195 not related to HAPA)	Yes (32)

## Abbreviations

CT: Computed tomography
CTA: Computed tomography angiography
DBD: Donation after brain death
DCD: Donation after cardiac death
HAPA: Hepatic artery pseudoaneurysm
ICU: Intensive care unit
IR: Interventional radiology
MELD: Model for end stage liver disease
MRI: Magnetic resonance imaging
OLT: Orthotopic liver transplantation
POD: Postoperative day
SBP: Spontaneous bacterial peritonitis
SLK: Simultaneous liver kidney transplant
US: Ultrasonography.
